# Treatment of Adolescent Idiopathic Scoliosis with the Conservative Schroth Method: A Randomized Controlled Trial

**DOI:** 10.3390/healthcare13060688

**Published:** 2025-03-20

**Authors:** Vanja Dimitrijević, Bojan Rašković, Miroslav P. Popović, Dejan Viduka, Siniša Nikolić, Nikola Jevtić, Samra Pjanić, Borislav Obradović

**Affiliations:** 1 Faculty of Sports and Physical Education, University of Novi Sad, 21000 Novi Sad, Serbia; dimitrijevicvanja@gmail.com (V.D.); boriscons@yahoo.com (B.O.); 2 Technical Faculty, Singidunum University, 11000 Belgrade, Serbia; miroslav.popovic@singidunum.ac.rs; 3 Faculty of Information Technologies, University Alfa BK, 11000 Belgrade, Serbia; dejan.viduka@alfa.edu.rs; 4 Institute of Physical Medicine, Rehabilitation and Orthopedic Surgery “Dr Miroslav Zotović”, 78000 Banja Luka, Bosnia and Herzegovina; sinisamnikolicbl@gmail.com (S.N.); samra.pjanic@hotmail.com (S.P.); 5 Faculty of Medicine, Department of Physiotherapy, University of Banja Luka, 78000 Banja Luka, Bosnia and Herzegovina; 6 Scolio Centar, 21000 Novi Sad, Serbia; njevticns@gmail.com

**Keywords:** Schroth method, adolescent idiopathic scoliosis, Cobb angle, randomized controlled trial

## Abstract

**Objective:** The objective of this study was to determine the effectiveness of the conservative Schroth method in patients with adolescent idiopathic scoliosis. **Methods:** A total of 34 respondents, 24 male and 10 female, aged between 11 and 16 years, participated in the research. The study was a single-blind randomized trial, in which subjects were divided into control and experimental groups by stratified randomization according to the stratum of the Cobb angle. The control group performed the Schroth method at home without the supervision of Schroth therapists, while the experimental group performed the Schroth method under the supervision of Schroth therapists three times a week for 90 min over eight weeks. Initial and final outcome measurements were performed: Cobb angle, angle of trunk rotation, vital capacity, forced vital capacity, forced expiratory volume in the first second, the percentage of forced expiratory volume in the first second in forced vital capacity, and chest expansion. **Results:** There was a statistically significant improvement in all measured outcomes in the experimental group, while no statistically significant changes were recorded in the control group. The Cobb angle decreased by 2.12°, while ATR decreased by 2.88°; VC increased by 0.15 L, FVC by 0.13 L, FEV1 by 0.1 L, and CE increased by 0.78 cm. **Conclusions:** The application of an eight-week therapy program using Schroth method by subjects with adolescent idiopathic scoliosis had statistically significant changes in all measured outcomes in the group that was supervised by Schroth’s therapists, while there was no statistically significant improvement in the group that applied therapy at home without supervision.

## 1. Introduction

Idiopathic scoliosis is a complex deformity of the spine that develops three-dimensionally and results in the appearance of frontal curves, fixed rotations of the vertebrae, and flattening of the sagittal physiological curves [1]. When scoliosis develops between 10 years of age and the end of growth, it is called adolescent idiopathic scoliosis (AIS) [2]. Despite extensive clinical, epidemiological, and basic science research, the etiopathogenesis of AIS remains unknown [3]. AIS is often seen in multiple members of the same family, strongly suggesting that it has a genetic component [4]. The prevalence of AIS is 2% to 3% in the general population, of which nearly 10% require some form of treatment, and up to 0.1% of these will require surgery [5]. When a diagnosis of scoliosis is made, the primary concern is whether there is an underlying cause and whether the curve will progress. The three main determinants of progression are the gender of the patient, future growth potential, and the size of the curve at the time of diagnosis [6,7,8]. The greater the growth potential and the greater the curve, the greater the likelihood of curve progression. The magnitude of the curve tends to increase throughout the lifespan, but the degree of progression over the lifetime and time at risk vary depending on many factors [9,10]. The Risser score (from 0 to 5) provides a useful estimate of how much skeletal growth remains by assessing the progress of bony fusion of the iliac apophysis [11]. In a study [12], the Risser score was directly correlated with the risk of curve progression. The risk of curve progression can also be assessed by taking into account the gender of the patient and the time of menarche. This information does not ultimately determine whether a particular curve will progress, but only the general risk of curve progression [13,14].

Early diagnosis of scoliosis is essential to initiate a timely and appropriate treatment, preventing potential respiratory, psychological, and social complications associated with the condition [15]. Depending on the magnitude of the curve, treatment approaches consist of different types of exercises, wearing a brace, and surgery for the deformity caused by AIS [16]. Stopping the progression of the curve in puberty, preventing or treating respiratory dysfunction, preventing or treating spinal pain syndrome, and improving aesthetics through postural correction are the main goals of applying various conservative treatments [9]. The most commonly used conservative methods are: Schroth method, scientific exercise approach to scoliosis (SEAS), Lyon method which is combined with Lyon brace, and core stabilization exercises [17,18,19,20,21].

The Schroth method was developed by Katharina Schroth in 1920 and has been continuously improved through the treatment of approximately 3000 cases of scoliosis per year. The principles of active 3D posture correction, corrective breathing and correction of postural perception, form the basis of what is known as the Schroth method as a treatment of scoliosis [22]. The method aims to derotate, elongate, and stabilize the spine through individualized exercises tailored to the patient’s specific curve pattern. A key component of the Schroth method is rotational angular breathing, a specialized technique that encourages expansion of the concave side of the ribcage, which is typically compressed due to scoliosis. Combined with targeted postural adjustments, this breathing technique helps improve lung function, rib mobility, and overall spinal alignment. Additionally, Schroth therapy places a strong emphasis on sensorimotor training, helping patients develop a conscious awareness of their spinal position to actively maintain a corrected posture in daily activities. Schroth’s classification system is derived from Schroth’s principle of dividing the body into blocks [23]. According to Schroth’s classification system, different types of scoliosis always start with a large curve followed by relevant secondary curves [22]. The exercises are customized based on the specific curve pattern, ensuring an individualized approach to treatment. There are numerous studies that confirm the positive effects in the treatment of idiopathic scoliosis using the Schroth method [18,24,25,26,27,28,29,30,31,32]. The primary goal of this study is to evaluate the effectiveness of the Schroth method as a conservative treatment for AIS by assessing its impact on key clinical and functional parameters. Specifically, we aim to determine whether the structured application of Schroth exercises can lead to reduction in spinal curvature (Cobb angle), improvement in trunk alignment (angle of trunk rotation, ATR), and enhancement of pulmonary function (vital capacity, VC; forced vital capacity, FVC; forced expiratory volume in one second, FEV1; and the FEV1/FVC ratio). Additionally, we assess the method’s influence on chest expansion (CE) as an indicator of thoracic mobility and respiratory efficiency. A secondary objective is to compare the effectiveness of supervised Schroth therapy (performed under the guidance of a trained therapist in a clinical setting) versus self-administered home-based Schroth exercises. By examining differences in treatment outcomes between these two approaches, this study seeks to determine whether guided professional supervision leads to superior therapeutic benefits compared to independent practice at home. Based on prior research and clinical experience, we hypothesize that the Schroth method will significantly improve scoliosis-related parameters in AIS patients, leading to: a reduction in Cobb angle, indicating structural improvement in spinal alignment; a decrease in the ATR reflecting better postural symmetry; an increase in VC, FVC, and FEV1, demonstrating improved respiratory function; enhanced CE, suggesting greater thoracic mobility and breathing efficiency. Supervised Schroth therapy will yield superior results compared to self-administered home-based exercises, as professional guidance ensures: more precise execution of exercises with real-time corrections; greater adherence to the prescribed treatment regimen; and improved neuromuscular engagement and postural re-education, leading to more effective long-term results.

## 2. Materials and Methods

### 2.1. Study Design

The study was designed as a randomized, single-blind, 1:1 parallel-group trial conducted at Scolio Centar in Novi Sad between March and June 2023. Ethical approval was obtained from the Ethics Committee of the Faculty of Sports and Physical Education at the University of Novi Sad (Approval No: 49-02-04/2023-1). Prior to participation, informed consent was obtained from the parents of all participants. The study included children diagnosed with adolescent idiopathic scoliosis (AIS), aged between 10 and 18 years, with a Cobb angle greater than 10 degrees and a Risser sign between 0 and 5. Participants who had previously undergone any form of scoliosis treatment were excluded from the study. Additional exclusion criteria included the presence of contraindications for exercise, psychological disorders, neuromuscular diseases, and a history of spinal surgery. Throughout the study, participants did not use braces.

### 2.2. Sample Size and Randomization

The sample size calculation was conducted using G*Power software (version 3.1.9.7, Heinrich-Heine-Universität Düsseldorf, Düsseldorf, Germany) [33]. To achieve a statistical power of 80% (1 − β error probability) at a significance level of α = 0.05, a repeated-measures analysis of variance (ANOVA) was performed. Based on the expected moderate effect size of 0.3, the required sample size was estimated at 24 participants. However, to enhance statistical power and reliability, the study included as many eligible participants as possible without violating the methodological framework, resulting in a total of 34 subjects. Participants were randomly assigned to one of two groups: an experimental group that received supervised Schroth therapy in a clinical setting and a control group that performed Schroth exercises at home without direct supervision. The randomization process was stratified based on the Cobb angle and executed using Microsoft Excel (2016). Allocation concealment was ensured by using sealed envelopes that were opened only at the moment of participant enrollment. To maintain objectivity, all baseline and post-treatment assessments were conducted by a blinded investigator (B.O.), who was unaware of the group assignments throughout the study.

### 2.3. Outcome Measurements

The primary outcome measure in this study was the Cobb angle, which was assessed using a standard anterior–posterior standing radiograph of the entire spine [34]. The angle of trunk rotation (ATR) was measured with a Bunnell scoliometer while the participant was in a forward bending position, with the device placed centrally over the spine. The ATR is expressed in degrees [35]. Pulmonary function was assessed by measuring vital capacity (VC), forced vital capacity (FVC), and forced expiratory volume in one second (FEV1) using a Spirolab portable spirometer (MIR, Rome, Italy). These pulmonary measurements were performed in an upright position, with each test repeated three times and the average value recorded. Additionally, chest expansion was evaluated manually with a tape measure positioned at the junction between the xiphoid process and the sternum.

### 2.4. Interventions

The intervention period lasted eight weeks and included a total of 24 sessions. Participants in the experimental group performed supervised Schroth exercises under the guidance of a certified physiotherapist (N.J.), attending 90 min training sessions three times per week. These exercises were specifically designed to improve trunk symmetry and included a combination of stretching, flexion, spinal elongation, derotation, strengthening, and rotational breathing techniques [22]. The control group, on the other hand, received initial instructions from the same physiotherapist on how to perform Schroth exercises independently at home. They were encouraged to practice as consistently as possible, following the prescribed regimen. The full details of the Schroth exercise program are provided in Appendix A.

### 2.5. Statistical Analysis

Statistical analysis was conducted using SPSS Statistics for Windows (version 26.0, IBM Corp., 2010, Armonk, NY, USA). The normality of data distribution was assessed using the Shapiro–Wilk test, confirming that all measured outcomes followed a normal distribution. To compare baseline characteristics between the experimental and control groups, an independent T-test was used for continuous variables, while categorical variables were analyzed using Pearson’s chi-square test. For ordinal variables, the Mann–Whitney test was applied. To evaluate changes over time and differences between groups, a mixed-model ANOVA was employed. Prior to conducting ANOVA, two key assumptions were tested: Levene’s test for equality of variance and Box’s M test for equality of covariance. The assumption of equal covariances was not met for the VC outcome, and neither assumption was met for the FEV1/FVC ratio. Consequently, Pillai’s trace, a more robust alternative to Wilks’ lambda, was utilized for these analyses [36]. The effect sizes were reported using partial eta squared (η^2^), with threshold values set at 0.10 for small, 0.25 for medium, and 0.40 for large effect [37]. The level of statistical significance was set at *p* < 0.05 for all analyses.

## 3. Results

**Figure 1:**
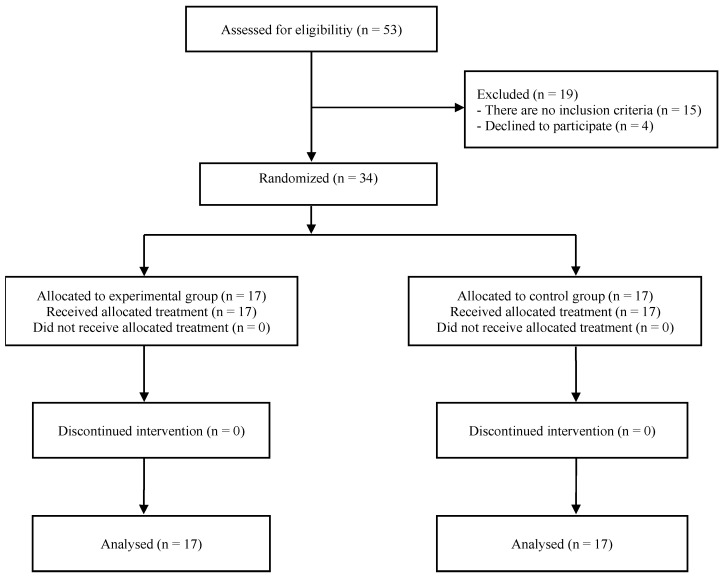
Flow diagram of the participants.

Fifty-three respondents who had scoliosis were admitted to the department, and forty-three respondents met the conditions to be included in the research. After randomization, patients were assigned n = 17 to EG and n = 17 to CG. All subjects who started the study completed the intended treatment in full. The research flow diagram is shown in Figure 1. In a sample of 34 subjects randomly distributed in EG and CG, there was basically no significant difference between the groups in age, weight, Cobb angle, and Risser sign. There was a statistically significant difference in height and body mass index (Table 1). For the Cobb angle, the interaction between group and time showed an effect size of η^2^ = 0.57, which, according to Cohen’s guidelines, represents a large effect size (Table 2). This indicates a meaningful improvement in spinal curvature in the experimental group, suggesting that the Schroth method is highly effective in correcting spinal deformities compared to the control group. A significant separate (basic) influence of time was also determined, F = 34.51; *p* = 0.000; η^2^ = 0.52. Regarding the ATR, the effect size of η^2^ = 0.61 also falls within the large effect size category (Table 2). This substantial reduction in ATR in the experimental group implies that the Schroth method significantly enhances spinal alignment and posture. A significant separate (basic) influence of time was also determined, F = 46.43; *p* = 0.000; η^2^ = 0.59. For respiratory function outcomes, the results were similarly impactful. Vital capacity (VC): The effect size of η^2^ = 0.53 is considered large, indicating that the Schroth method substantially improves lung capacity (Table 2). A significant separate (basic) influence of time was also determined, F = 50.82; *p* = 0.000; η^2^ = 0.61. Forced vital capacity (FVC): With an effect size of η^2^ = 0.66, this outcome also shows a large effect, suggesting significant benefits to respiratory efficiency (Table 2). A significant separate (basic) effect of time was also determined, F = 78.66; *p* = 0.000; η^2^ = 0.71. Forced expiratory volume in one second (FEV1): The large effect size of η^2^ = 0.67 supports that the Schroth method effectively enhances respiratory muscle strength (Table 2). A significant separate (basic) influence of time was also determined, F = 73.86; *p* = 0.000; η^2^ = 0.7. Chest expansion (CE): The effect size of η^2^ = 0.78 is well above the threshold for a large effect, highlighting the substantial positive impact of the Schroth method on thoracic mobility and respiratory function (Table 2). A significant separate (basic) influence of time was also determined, F = 164.74; *p* = 0.000; η^2^ = 0.84. In contrast, the FEV1/FVC ratio showed no significant interaction, with an effect size of η^2^ = 0.05, which Cohen’s guidelines classify as no effect (Table 2). This suggests that while the Schroth method improves individual respiratory measures, it does not significantly influence the ratio between FEV1 and FVC.

## 4. Discussion

The results of our research show compliance with the set hypothesis that the application of the Schroth method under the supervision of a physiotherapist in clinical conditions has positive effects in slowing down and stopping scoliosis, reducing the Cobb angle, ATR, and improving pulmonary function. The Schroth method, under the supervision of a physiotherapist in clinical conditions, has better effects than the application of the same method in home conditions.

In the treatment of AIS as a pathology of unknown cause, surgical and non-surgical (conservative) methods are used. Despite the great advances in technology and science, operative approaches to treatment are not completely safe, so the risks of side effects can often lead to abandoning this type of treatment. Timely detection of scoliosis is crucial for preventing serious complications, with rehabilitation specialists playing a vital role in halting the progression of the condition [15]. Of all the conservative methods, the Schroth method has the largest number of published studies. Unlike other scientific fields, the amount of such research is still relatively small, and with this research, we tried to make a small contribution, so that the effects of applying conservative methods (primarily the Schroth method) in the treatment of AIS could be seen in an even better way. The main clinical and prognostic indications in the study of AIS are the Cobb angle and ATR [38]. All approaches and strategies that slow the progression of scoliosis and prevent the need for surgery are important in the treatment of AIS [39]. In our research, the Cobb angle decreased only in the experimental group by 2.12° (from 30.18 ± 8.19° to 28.06 ± 7.99°), which was recorded as statistically significant. Our results are similar to the results of the study [18], which conducted a six-month treatment and recorded the greatest reduction in the Cobb angle in the first six weeks of treatment. This study applied the same weekly intensity as our study. The study by [27], which was the only one like ours to use an eight-week program with three sessions per week, but each lasting 60 min, reported a 2.69° reduction in the Cobb angle in seven subjects, which largely coincides with our results. The study by [40] recorded a reduction in the Cobb angle by 2.65°, after an intensive six-week program carried out in five weekly sessions, and the results of this study largely coincide with our results. Other studies [26,27,28,29,30] report a generally greater reduction in the Cobb angle, and their results do not match our results. A study [41] that also applied an eight-week treatment with core stabilization exercises recorded similar results to ours, while other studies [42,43] that applied this method recorded greater reductions in the Cobb angle. Regarding other exercise-based conservative methods, the study [44] reports similar results to ours, while studies [45] report greater reductions in the Cobb angle. ATR, as the second most important indicator of idiopathic scoliosis and as the first diagnostic procedure, statistically significantly decreased only in EG, by 2.88° (from 11.24 ± 4.18° to 8.35 ± 3.18°). Our results almost completely coincide with the results of a study [30] that reports a reduction in ATR by 2.82°, while the results of other studies [24,28,46] have greater or lesser differences. In curves involving the thoracic spine, the mobility of the chest wall is reduced, and secondary effects of reduced spinal flexibility appear as a disorder [47,48]. VC is inversely correlated with the amount of curvature up to a Cobb angle of 10°. Even when resting VC is found to be normal, reduced exercise capacity is demonstrated even in children with mild curvatures (Cobb angle 5–20°) [49]. With the Schroth method, progress is made through rotational breathing techniques, where inhaled air is directed toward the concave sides of the chest and ribs to lengthen and mobilize the soft tissues in these areas [49]. This process provides the patient with proprioceptive enhancement of the sensation of spinal derotation and also helps to eliminate further inhalation of the convexity. This principle is an essential component of the Schroth method because the muscles in the convex areas are overstretched and unable to retract properly during exhalation [46]. In our research, the aforementioned was confirmed by recording a decrease in the Cobb angle and ATR, followed by an increase in VC, only in EG, by 0.16 l (from 2.81 ± 0.66 to 2.97 ± 0.65), which in our case recorded as a statistically significant increase. A study by [40] noted the same increase in VC after the first six weeks of intensive Schroth therapy. Of the other conservative exercise-based methods, only one study [45], examines this outcome and records a greater increase than our study. VC is a very suitable index for determining restrictive pulmonary function [50] but it is a rarely used outcome in published research. Just as VC is negatively correlated with the size of the curve, so is FVC, another important respiratory parameter, negatively correlated with the size of the curve in AIS [51]. With a greater degree of curvature, there is greater decrease in respiratory function as measured by FVC [52]. The study [53] confirms this correlation. In our case, the increase in FVC was statistically significant only in EG. At the initial measurement, the subjects had an average value of 2.51 L, and at the end of the eight-week treatment 2.63 L, which represents a difference of 0.12 L. The study by [46] recorded an improvement in FVC results by 0.10 L after applying the treatment. The study by [27] recorded better results of FVC by 0.33 L after the application of the Schroth method, which was followed by additional respiratory exercises using the SpiroTiger device (Idiag, Fehraltorf, Switzerland). Using other exercise-based methods, studies [45,54] report greater improvements in this outcome than ours. The recorded reduction of the Cobb angle and ATR, as in the case of VC, led to better results in the final measurement. As in the case of VC and FVC, the ratio of FEV1 is negatively correlated with the size of the curve, which means that a higher degree of the curve is accompanied by a lower value of FEV1 [52]. It is certain that the higher position of the curve reduces all the examined respiratory parameters to a greater degree, which is consistent with what the previously mentioned study stated—that there are greater deficits in patients with a thoracic curve. When idiopathic scoliosis affects the thoracic region, it can cause a rotational deformity that affects the shape and position of the chest. This, in turn, can affect breathing mechanics and potentially lead to reduced pulmonary function. Decreased pulmonary capacity can lead to a decrease in FEV1. In our research, FEV1 increased statistically significantly after treatment only in the experimental group by 0.1 l (from 2.2 ± 0.49 to 2.3 ± 0.48). The study by [46] recorded an improvement in FEV1 results by 0.09 L after applying the treatment. The study by [27] recorded better results of FEV1 by 0.39 L after the application of the Schroth method, which was followed by additional respiratory exercises. As for the previous outcome, studies [45,54] record better results in this outcome as well. As with the previous respiratory parameters, FEV1 increased after the reduction of the Cobb angle and ATR, and in this case, confirmed a negative correlation in relation to the size of the curve. The FEV1/FVC ratio, in the context of idiopathic scoliosis, may be relevant in cases where the curvature of the spine affects the thoracic region and potentially affects pulmonary function. Severe scoliosis can lead to changes in the shape and position of the chest, which can limit pulmonary expansion and compromise respiratory function. In our research, the FEV1/FVC ratio decreased statistically significantly only in the experimental group by 0.01, that is, by 1% (from 0.88 ± 0.01 to 0.87 ± 0.02). In the study by [27], FEV1/FVC ratio increased by 3.76%. Chest expansion (CE) is an important measurement in the evaluation of scoliosis because it provides valuable information about the effect of spinal curvature on respiratory function. AIS, especially when it affects the thoracic region, can lead to changes in the shape and position of the chest, potentially limiting pulmonary expansion during breathing. In our research, CE increased statistically significantly only in the experimental group by 0.78 cm, (from 6.26 ± 0.89 to 7.04 ± 0.84). In the study by [46], CE increased by 1.31 cm. The study by [55] records an increase in CE by 1.27 cm. Limited chest expansion may indicate reduced pulmonary capacity and reduced respiratory function. As scoliosis progresses and the curvature of the spine becomes greater, there may be a restriction of the space available for pulmonary expansion, leading to a decrease in pulmonary volume and potential respiratory limitations. Monitoring chest expansion over time can help healthcare providers monitor changes in respiratory function and determine the progression of respiratory impairment associated with AIS. The number of studies with which we compared our results highlights the limited number of studies that examined pulmonary function in patients with idiopathic scoliosis. The results we obtained in our research are in agreement with the results of our meta-analysis [56,57], which confirmed the positive effects of the Schroth method on patients with idiopathic scoliosis.

### 4.1. Clinical Implications

Reduction in spinal deformity: The experimental group showed significant decreases in the Cobb angle compared to the control group, emphasizing the effectiveness of the Schroth method in mitigating scoliosis progression. This is crucial for reducing the need for surgical interventions. Improvement in trunk alignment: significant improvements in the ATR were observed in the EG, suggesting enhanced postural symmetry and better cosmetic outcomes, essential for patient confidence and quality of life. Respiratory function enhancement: substantial improvements in VC, FVC, and FEV1 indicate the positive impact of the Schroth method on respiratory health, which is often compromised in AIS due to thoracic deformities.

Limited impact on FEV1/FVC ratio: the lack of significant changes in the FEV1/FVC ratio suggests that while overall respiratory capacity improved, relative airway function remained stable. Improved chest expansion: chest expansion (CE) significantly improved in the EG, reflecting enhanced thoracic mobility and better functional capacity, crucial for physical activity and overall well-being. Feasibility and adherence: the completion rate of 100% in both groups highlights the practicality and acceptability of the Schroth method for adolescents, making it a viable long-term treatment option.

### 4.2. Recommendations for Future Practice

Integration into routine care: the Schroth method should be incorporated into standard conservative management protocols for AIS to improve spinal alignment and prevent progression. Focus on early intervention: initiating the Schroth method early in the disease process may yield better Cobb angle reduction, trunk rotation, and respiratory function outcomes. Holistic treatment approach: combining the Schroth method with other conservative treatments, such as bracing or physical therapy, may optimize outcomes and address both structural and functional impairments in AIS. Tailored interventions: individualized treatment plans should account for differences in height, body mass index, and severity of scoliosis to maximize the method’s effectiveness. Long-term follow-up: future clinical practice should include long-term monitoring of patients to assess the durability of improvements in spinal curvature, respiratory function, and quality of life. Expanded research: further studies should explore the long-term impacts of the Schroth method, its effectiveness in severe scoliosis cases, and its integration with other therapeutic modalities. Development of guidelines: Evidence-based clinical guidelines should be developed to standardize the application of the Schroth method across healthcare settings.

### 4.3. Strengths and Limitations

The randomized controlled design ensured a high level of evidence by minimizing bias and improving the reliability of the results. Supervised Schroth therapy allowed for standardized execution of exercises, ensuring adherence and maximizing treatment effectiveness. Comprehensive outcome assessment, including spinal alignment (Cobb angle, angle of trunk rotation) and respiratory function (vital capacity, forced vital capacity, forced expiratory volume in one second, chest expansion), provided a holistic evaluation of the Schroth method’s impact. Complete follow-up with no dropouts increased the validity of the findings, eliminating concerns about attrition bias. This study has several limitations. First, the number of subjects included in the study is relatively small. Second, although the biggest changes were noted in the first weeks of treatment, we could say that eight weeks of treatment is still a short period. Third, after eight weeks of treatment, there was no follow-up period to quantitatively record further changes. Fourth, the lack of a home exercise group means that the study does not address the effectiveness of unsupervised Schroth exercises compared to supervised therapy. Fifth, the absence of a brace-wearing comparison group could provide insights into whether Schroth therapy alone is sufficient for cases requiring conservative management. Our research is the first such research conducted in Serbia, so we hope to correct all these shortcomings in our next research.

## 5. Conclusions

The results of this randomized controlled trial demonstrate that the Schroth method is an effective conservative treatment for adolescent idiopathic scoliosis, leading to significant improvements in spinal curvature, postural alignment, and respiratory function compared to the control group. Participants in the experimental group, who performed Schroth exercises under therapist supervision, showed a statistically significant reduction in the Cobb angle (*p* = 0.000) and angle of trunk rotation (*p* = 0.000), while no significant changes were observed in the control group. This confirms the corrective effect of Schroth-specific exercises on three-dimensional spinal deformity. Furthermore, the Schroth method significantly improved pulmonary function parameters, including vital capacity (*p* = 0.000), forced vital capacity (*p* = 0.000), and forced expiratory volume in one second (*p* = 0.000), suggesting that targeted postural and breathing exercises enhance respiratory mechanics in scoliosis patients. However, the FEV1/FVC ratio did not show significant changes, indicating that while lung volume and expansion improved, the airflow ratio remained stable. A significant increase in chest expansion (*p* = 0.000) was observed in the experimental group, reinforcing the effectiveness of Schroth-based breathing techniques in improving thoracic mobility and lung capacity. Importantly, all participants completed the intervention, demonstrating high feasibility and adherence to the program. These findings suggest that supervised Schroth therapy is more effective than unsupervised or standard care in managing adolescent idiopathic scoliosis, supporting its integration into conservative treatment protocols (ClinicalTrials.gov ID: NCT06877806).

## Figures and Tables

**Table 1:** Demographic and baseline clinical characteristics.

Characteristic	CG (n = 17)	EG (n = 17)	Comparison
			*p*
Age (years)	13.41 ± 1.63	14.11 ± 1.02	0.14
Height (cm)	163.12 ± 11.47	170.94 ± 8.52	0.03
Weight (kg)	53.32 ± 11.21	53.26 ± 8.02	0.99
Body mass index (kg/m^2^)	19.87 ± 2.66	18.16 ± 1.98	0.04
Gender (male/female)	5 (12)	5 (12)	1
Risser sign	1.65 ± 1.45	2 ± 1.28	0.21
Cobb angle	30.24 ± 6.51	30.18 ± 8.19	0.98
ATR	10.06 ± 2.97	11.24 ± 4.18	0.35
VC	2.69 ± 0.55	2.81 ± 0.66	0.55
FVC	2.36 ± 0.49	2.51 ± 0.56	0.43
FEV1	2.02 ± 0.38	2.2 ± 0.49	0.23
FEV1/FVC	0.86 ± 0.03	0.88 ± 0.01	0.01
CE	6.13 ± 0.78	6.26 ± 0.89	0.65

CG: control group; EG: experimental group; ATR: angle of trunk rotation; VC: vital capacity; FVC: forced vital capacity; FEV1: forced expiratory volume at one second; CE: chest expansion.

**Table 2:** Summary of findings for group–time interaction.

Outcomes	Group	Pre-Test	Post-Test	Difference Value	Group × Time Interaction
		Mean ± SD	Mean ± SD	Mean ± SE	
Cobb angle	CG (n = 17)	30.24 ± 6.51	30.35 ± 6.26	−0.12 ± 0.21	F = 43.1; *p* = 0.000; η^2^ = 0.57
	EG (n = 17)	30.18 ± 8.19	28.06 ± 7.99	2.12 ± 0.27	
ATR	CG (n = 17)	10.06 ± 2.97	10.12 ± 2.78	−0.06 ± 0.2	F = 50.38; *p* = 0.000; η^2^ = 0.61
	EG (n = 17)	11.24 ± 4.18	8.35 ± 3.18	2.88 ± 0.36	
VC	CG (n = 17)	2.69 ± 0.55	2.7 ± 0.55	−0.01 ± 0.01	F = 35.69; *p* = 0.000; η^2^ = 0.53
	EG (n = 17)	2.81 ± 0.66	2.97 ± 0.65	−0.15 ± 0.02	
FVC	CG (n = 17)	2.36 ± 0.49	2.37± 0.47	−0.01 ± 0.01	F = 62.77; *p* = 0.000; η^2^ = 0.66
	EG (n = 17)	2.51 ± 0.56	2.63 ± 0.55	0.13 ± 0.01	
FEV1	CG (n = 17)	2.02 ± 0.38	2.02 ± 0.37	−0.00 ± 0.00	F = 65.33; *p* = 0.000; η^2^ = 0.67
	EG (n = 17)	2.2 ± 0.49	2.3 ± 0.48	−0.1 ± 0.01	
FEV1/FVC	CG (n = 17)	0.86 ± 0.03	0.86 ± 0.03	0.00 ± 0.00	F = 1.71; *p* = 0.2; η^2^ = 0.05
	EG (n = 17)	0.88 ± 0.01	0.87 ± 0.02	0.01 ± 0.00	
CE	CG (n = 17)	6.13 ± 0.78	6.21 ± 0.77	−0.08 ± 0.03	F = 110.96; *p* = 0.000; η^2^ = 0.78
	EG (n = 17)	6.26 ± 0.89	7.04 ± 0.84	−0.78 ± 0.06	

CG: control group; EG: experimental group; ATR: angle of trunk rotation; VC: vital capacity; FVC: forced vital capacity; FEV1: forced expiratory volume at one second; CE: chest expansion; SD: standard deviation; SE: standard error.

## Data Availability

Any personal or patient data are unavailable due to privacy or ethical restrictions. All other data are available from the corresponding author upon reasonable request.

## Abbreviations

The following abbreviations are used in this manuscript:

IS	Idiopathic scoliosis
AIS	Adolescent idiopathic scoliosis
CE	Chest expansion
CG	Control group
FVC	Forced vital capacity
FEV1	Forced expiratory volume in 1 s
ATR	Angle of trunk rotation
VC	Vital capacity
SEAS	Scientific exercises approach to scoliosis

## Appendix A

The following table presents the Schroth experimental program used in the study. This structured program was implemented in the experimental group under the supervision of Schroth therapists.

**Table A1:** Schroth Experimental Program.

Exercise Name	Position	Description (Optional)
Warm-up	-	General mobilization and activation
Side bending	Sitting	Active lateral flexion for scoliosis correction
Derotation of the ventral prominence	Supine	Rotation correction of the prominent ribcage
Shoulder Counter Traction	Supine	Shoulder stabilization exercise
Shoulder Counter Traction	Side	Shoulder traction from a lateral position
Shoulder Counter Traction	Prone	Posterior shoulder traction engagement
Between two poles	Sitting	Spine elongation and postural awareness
Muscle cylinder kneeling	Kneeling	Core engagement and stability training
Side plank small	Side-lying	Core and oblique muscle activation
Dead bug	Supine	Core stabilization with limb movement
Final stretching	-	Flexibility and relaxation exercises
